# Serum BDNF Levels in Relation to Illness Severity, Suicide Attempts, and Central Serotonin Activity in Patients with Major Depressive Disorder: A Pilot Study

**DOI:** 10.1371/journal.pone.0091061

**Published:** 2014-03-24

**Authors:** Young-Min Park, Bun-Hee Lee, Tae Hyun Um, Sollip Kim

**Affiliations:** 1 Department of Psychiatry, Ilsan Paik Hospital, Inje University College of Medicine, Goyang, Republic of Korea; 2 Department of Psychiatry, Gangnam Eulji Hospital, Eulji University, Seoul, Republic of Korea; 3 Department of Laboratory Medicine, Ilsan Paik Hospital, Inje University College of Medicine, Goyang, Republic of Korea; Max Planck Institute of Psychiatry, Germany

## Abstract

The aim of this study was to test the hypothesis that serum levels of brain-derived neurotrophic factor (BDNF) are correlated with the loudness dependence of auditory evoked potentials (LDAEP). The question of whether there is a difference in BDNF levels between depressive patients according to their illness severity, history of suicide attempts, and central serotonin activity was also addressed. A sample of 51 patients who met the criteria for major depressive disorder following diagnosis using axis I of the fourth edition of the Diagnostic and Statistical Manual of Mental Disorders – text revision comprised the study subjects. The patients were stratified into two subgroups based on their illness severity, history of suicide attempts, and their LDAEP values. The LDAEP was evaluated by measuring the auditory event-related potentials, and serum BDNF was measured using blood sampling before beginning medication with serotonergic agents. There was no difference in serum BDNF levels between the two patient subgroups. The subgroup with moderate-to-severe depression (*n* = 16) was reanalyzed after stratifying it into two subgroups according to LDAEP and BDNF values (dichotomized at the medians into low and high). The high-LDAEP subgroup had higher serum BDNF levels and total Barratt Impulsiveness Scale score than the low-LDAEP subgroup (*p* = 0.03 and 0.036, respectively). Serum BDNF levels were positively correlated with LDAEP and total Beck Hopelessness Scale (BHS) score (*r* = 0.56, *p* = 0.025, and *r* = 0.59, *p* = 0.016, respectively). The high-BDNF subgroup had a higher LDAEP and total BHS score than the low-BDNF subgroup (*p* = 0.046 and *p* = 0.011, respectively). This is the first study to demonstrate a relationship between the BDNF level and LDAEP in Asian depressive patients. Intriguingly, the high-BDNF subgroup (divided according to illness severity) exhibited a more severe psychopathology on some psychometric rating scales, a finding that conflicts with previous results.

## Introduction

Brain-derived neurotrophic factor (BDNF) is considered a valid indicator of depressive state. Clinical studies have indicated that serum or plasma BDNF levels are decreased in patients with untreated major depressive disorder (MDD), and that antidepressant treatment can restore the decreased BDNF level to the normal value [1]. Lee and colleagues found that BDNF levels were significantly lower in MDD patients with recurrent episodes than in MDD patients with a first episode or normal controls, and that BDNF levels were significantly lower in suicidal MDD patients than in their nonsuicidal counterparts [2]. There is also some evidence that levels of BDNF are positively correlated with those of serotonin. Some researchers have reported that BDNF promotes the sprouting of mature, uninjured serotonergic axons, and that chronic treatment with BDNF induces enhancement of the regenerative sprouting of neurotoxin (p-chloroamphetamine)-damaged serotonergic axons [3], [4], [5]. Lang and colleagues also found that serum BDNF levels are positively correlated with central serotonergic neurotransmission, using loudness dependence of auditory evoked potentials (LDAEP) [5]. However, their results have yet to be replicated.

The N1/P2 amplitude of auditory evoked potentials can increase with the intensity of the auditory stimulus. Serotonin usually plays an important role in suppressing the size of this increased N1/P2 response to increased auditory stimulus in order to minimize damage to the brain [6], thus leading to the hypothesis that strong serotonin activity can reduce this effect [7]. Furthermore, some investigators have considered this change in the N1/P2 amplitude in response to increasing the stimulus intensity to be the LDAEP [6], [7]. In addition, a weak LDAEP reflects high serotonergic neurotransmission in the central nervous system [8], [9]. There was some evidence that LDAEP can be a useful tool in patients with MDD [10], [11], [12], [13]. In addition, it was found that depressed patients with higher LDAEP showed better treatment response to SSRIs [7], [14], [15], [16], [17], [18]. Thus, it is possible for LDAEP to be a tool for evaluating central serotonergic activity in patients with MDD.

The aim of this study was to test the hypothesis that serum BDNF levels are correlated with LDAEP. The question of whether there is a difference in BDNF levels between depressive patients according to their illness severity, history of suicide attempts, and central serotonin activity was also addressed.

## Materials

### Subjects

In total, 77 outpatients aged between 18 and 65 years who met the Diagnostic and Statistical Manual of Mental Disorders (DSM-IV)–text revision criteria for MDD were enrolled from Ilsan Paik Hospital. The MDD diagnosis was determined in all subjects by trained psychiatrists. Subjects who had psychotic symptoms, any additional mental disorders on axis I or II of the DSM-IV, or major medical and neurological disorders were excluded in order to remove bias. None of the subjects had a history of hypomanic or manic episodes. Follow-up loss, withdrawals of consent, and insufficient data resulted in the final inclusion of 51 patients.

The baseline LDAEP was evaluated by measuring the auditory event-related potential, and serum BDNF was measured using blood sampling before beginning medication with serotonergic agents. In addition, several psychometric ratings were completed by the investigators at baseline. The relationship between LDAEP and suicidality or bipolarity has been evaluated previously in studies with a similar design [11], [19]. In the present study, BDNF measurement was added to the previous study design.

The cohort was stratified into two subgroups according to their illness severity [mild vs moderate or severe, Hamilton Depression Rating Scale (HAMD) score of >17], history of suicide attempts (yes vs no), and their LDAEP values (dichotomized at the median into low vs high), which had been used as a noninvasive biological marker of central serotonergic activity and treatment response [20], [21], [22]. The subgroup with moderate-to-severe depression was subjected to further analysis.

Depression severity was assessed using the clinician-administered 17-item HAMD scale [23]. Furthermore, the (BHS) [24], the Barratt Impulsiveness Scale (BIS) [25], the Hamilton Anxiety Scale (HAMA) [26], and the Beck Scale for Suicidal Ideation (BSS) [27] were applied. Validation studies were conducted for all of these Korean version scales except HAMA, which demonstrated their validity and reliability as good psychometric tools [28], [29], [30], [31].

The study protocol was approved by the ethics committee of Inje University Ilsan Paik Hospital, and written informed consent to participate was obtained from all patients before beginning the investigation.

### EEG methods

The potential confounding influences of drugs were minimized by measuring the LDAEP before treatment with antidepressants or serotonergic agents. The subjects who participated in our study were not allowed to have taken any psychotropic agent within 2 months before visiting the hospital, except for a hypnotic drug (benzodiazepine or zolpidem).

Each subject was seated in a chair in a sound-attenuated room. The auditory stimulation comprised 1000 stimuli with an interstimulus interval randomized to between 500 and 900 ms. Tones of 1000 Hz and 80-ms duration (with 10-ms rise and fall times) were generated by E-Prime software (Psychology Software Tools, Pittsburgh, PA, USA) and presented at five intensities (55, 65, 75, 85, and 95 dB SPL) via headphones (MDR-D777, Sony, Tokyo, Japan). EEG data were recorded from 64 scalp sites using silver/silver-chloride electrodes according to the international 10–20 system (impedance <10 kΩ), using an Auditory Neuroscan NuAmp amplifier (Compumedics USA, El Paso, TX, USA). Data were collected at a sampling rate of 1000 Hz, using a bandpass filter of 0.5–100 Hz. In addition, four electrodes were used to measure both horizontal and vertical electrooculograms.

Data were reanalyzed using Scan 4.3 software with a bandpass filter of 1–30 Hz, and ocular contamination was removed using standard blink-correction algorithms [32]. Event-related potential sweeps with artifacts exceeding 70 µV were rejected at all electrode sites. For each intensity and for each subject, the N1 peak (negative-most amplitude between 80 and 130 ms after the stimulus) and P2 peak (positive-most peak between 130 and 230 ms after the stimulus) were then determined at the Cz electrode. The peak-to-peak N1/P2 amplitudes were calculated for the five stimulus intensities, and the LDAEP was calculated as the slope of the linear-regression curve. The Cz electrode was chosen because previous studies have shown this to be a reliable site at which the amplitude is higher than at other electrode sites [12], [22], [33]. The dipole source analysis for the calculation of LDAEP has been used in several studies [9], [34], producing results similar to those obtained when using cortical activity [33]. Moreover, many LDAEP studies involving MDD, bipolar disorder, and anxiety disorder patients along with healthy controls have been conducted based on cortical activity [8], [35], [36], [37], [38], [39], [40]. Thus, the current study calculated the LDAEP from the cortical activity instead of using dipole source analysis. In addition, quality control was conducted periodically, such as checking the stimulus intensity (every week) and the impedance of the electrical cap (every day).

### Measurement of BDNF

Serum BDNF levels were measured using enzyme-linked immunosorbent assay (ELISA) kits (Quantikine Human BDNF, R&D Systems, Minneapolis, MN, USA) according to the manufacturer's instructions. Each assay was performed in duplicate. The actual concentration for each sample was calculated using the four-parameter fit logistic curve equation. The ELISA plate readings were conducted using a VersaMax microplate reader (Molecular Devices, Sunnyvale, CA, USA).

### Analysis

The demographic, psychopathological, and biological measures of the two groups were compared using Student's *t*-tests, Mann-Whitney U tests (which were used instead of *t*-tests when the Kolmogorov-Smirnov test revealed significant probability values in certain variables), and chi-square tests, and correlation analysis (Pearson's and Spearman's correlation) using SAS 9.3 and SALT 2.5 version. All tests were two tailed, and group differences were tested at the *p*<0.05 level.

## Results

There was no difference in serum BDNF levels between the two subgroups of MDD patients stratified according to illness severity (mild, *n* = 35, vs moderate and severe, *n* = 16), history of suicide attempts (no, *n*o = 33, vs yes, *n* = 18), and LDAEP values (dichotomized at the median into low, *n* = 26, vs high, *n* = 25; Table 1). Moreover, there was no correlation between the serum BDNF level and any of the measured variables, including LDAEP and psychometric rating except total BIS score (Table 2). However, reanalysis of the subgroup with moderate-to-severe illness severity (*n* = 16) revealed some positive findings. This group was stratified into two subgroups according to their LDAEP values (dichotomized at the median into low, *n* = 8, and high, *n* = 8) and reanalyzed using Mann Whitney test (Table 3). The serum BDNF levels and total BIS score were higher in the high-LDAEP subgroup than in the low-LDAEP subgroup (*p* = 0.03 and *p* = 0.036, respectively; Table 3). In addition, a tendency toward a higher total BHS score was found in the high-LDAEP subgroup (*p* = 0.092; Table 3). The total HAMD, HAMA, and BSS scores did not differ between these two subgroups (Table 3). Spearman correlation revealed a positive correlation between the serum BDNF level and LDAEP and total BHS score (*r* = 0.56, *p* = 0.025 and *r* = 0.59, *p* = 0.016, respectively; Figures S1 and S2). In addition, a marginal positive correlation was found between serum BDNF levels and total BIS score (*r* = 0.47, *p* = 0.067; Figure S3). Conversely, there was no correlation between the serum BDNF level and total HAMD, HAMA, and BSS scores.

**Table 1:** Brain-derived neurotrophic factor (BDNF) levels in the two subgroups divided according to illness severity (mild vs moderate or severe), history of suicide attempts (no vs yes), and loudness dependence of auditory evoked potentials (LDAEP) values (dichotomized at the median into low vs high).

Division criteria	BDNF (ng/ml) (*n* = 51)		*p*
Severity (mild vs moderate or severe)	22.44±9.8 (*n* = 35)	23.94±7.38 (*n* = 16)	0.59
History of suicidal attempts (no vs yes)	21.93±24.71 (*n* = 33)	24.71±7.7 (*n* = 18)	0.3
LDAEP values (low vs high)	21.54±9.45 (*n* = 26)	24.34±8.6 (*n* = 25)	0.27

Data are mean±SD values.

**Table 2:** Correlation (Pearson's coefficient) between BDNF levels and LDAEP or psychometric ratings.

	LDAEP	HAMD	HAMA	BIS	BHS	BSS
BDNF	0.1 (*p* = 0.47)	0.1 (*p* = 0.48)	0.011 (*p* = 0.94)	0.3 (*p* = 0.036)	0.23 (*p* = 0.1)	0.17 (*p* = 0.23)

HAMD, Hamilton Depression Rating Scale; HAMA, Hamilton Anxiety Scale; BIS, Barratt Impulsiveness Scale; BHS, Beck Hopelessness Scale; BSS, Beck Scale for Suicidal Ideation.

**Table 3:** Gender, age, BDNF levels, and psychometric ratings of patients with moderate or severe depression according to low and high LDAEP (dichotomized at the median).

	Low LDAEP (*n* = 8)	High LDAEP (*n* = 8)	*p*
Gender (M/F)^a^	3/5	1/7	0.57
Age (years)	37.0±14.03	40.75±14.05	0.79
BDNF (ng/ml)	20.01±6.69	27.8±6.14	0.03*
HAMD	21.0±3.34	21.5±4.24	0.79
HAMA	20.88±3.27	22.25±3.58	0.56
BIS	73.25±9.71	88.0±14.97	0.036*
BHS	11.38±6.05	15.75±6.54	0.092
BSS	13.25±10.87	19.25±10.14	0.25

*p<0.05.

a Chi-square test.

Except where stated otherwise, data are mean±SD values.

Further analysis of the subgroup with moderate-to-severe illness severity was performed after stratifying it into two further subgroups according to the serum BDNF level (dichotomized at the median to low, *n* = 8, and high, *n* = 8; Table 4). The LDAEP and total BHS score were higher in the high-BDNF subgroup than in the low-BDNF subgroup (*p* = 0.046 and *p* = 0.011, respectively; Table 4). In addition, there was a tendency toward a higher total BIS score in the high-BDNF subgroup (*p* = 0.093; Table 4). However, the total HAMD, HAMA, and BSS scores did not differ between the two subgroups (Table 4).

**Table 4:** Gender, age, LDAEP, and psychometric ratings of patients with moderate or severe depression according to low and high BDNF levels (dichotomized at the median).

	Low BDNF (*n* = 8)	High BDNF (*n* = 8)	*p*
Gender (M/F)^a^	2/6	2/6	1
Age (years)	39.88±12.44	37.88±15.66	0.64
LDAEP (µV/10 dB)	0.35±0.84	1.18±0.81	0.046*
HAMD	20.5±3.12	22.0±4.28	0.49
HAMA	21.25±3.37	21.88±3.60	0.31
BIS	74.88±7.64	86.38±17.57	0.093
BHS	9.75±6.90	17.38±3.07	0.011*
BSS	12.5±10.94	20.0±9.47	0.11

*p<0.05.

a Chi-square test.

Except where stated otherwise, data are mean±SD values.

## Discussion

The present study found no differences in the serum BDNF level between depressive patients stratified according to illness severity, history of suicide attempts, and LDAEP values (dichotomized at the median; Table 1). In addition, there was no correlation between the serum BDNF level and any of the measured variables, including the LDAEP and psychometric rating except total BIS score (Table 2). However, reanalysis of the subgroup with moderate-to-severe illness severity revealed some positive findings after stratifying it according to BDNF and LDAEP values (dichotomized at the medians into low and high). It was hypothesized that biological changes associated with BDNF levels are larger in patients with a greater severity of depression based on meta-analyses that have shown significant differences between the effects of antidepressants and placebo on changes in HAMD or MADRS scores in such patients [41], [42]. Thus, the sample was reanalyzed whilst excluding patients with mild depression.

Lee and colleagues found that plasma BDNF levels were significantly lower in suicidal MDD patients than in their nonsuicidal counterparts [2], and this finding was corroborated by Kim and colleagues [43]. The finding of the present study of no difference in serum BDNF levels between two subgroups divided according to history of suicide attempts (Table 1) is not consistent with the previous results [2], [43]. These conflicting results may be attributable to differences in the severity of depression between the subjects included in the studies, since those in the present study were all outpatients while those of Lee et al. [2] and Kim et al. [22] were not. Furthermore, BDNF levels were measured in different media between the studies (i.e., serum vs plasma). Moreover, the serum BDNF level has been found to be about 100-fold higher than the plasma BDNF level [44], [45]. This large difference originates from the clotting process releasing circulating BDNF stored in platelets [45], [46], [47].

The total BIS and BHS scores were higher in the high-LDAEP subgroup than in the low-LDAEP subgroup (Table 3). This indicates that depressed patients with low serotonin activity are more vulnerable to aggressive or impulsive behaviors, including suicidality, than are those with high serotonin activity. These results are consistent with previous results [19], [48], [49].

In the present study, the high-BDNF subgroup had a higher LDAEP – as indicated by lower central serotonergic activity – than the low-BDNF subgroup (Table 4). In addition, serum BDNF levels were positively correlated with LDAEP (Figure S1). Conversely, Lang and colleagues reported that serum BDNF levels were negatively correlated with LDAEP. However, there is a growing body of evidence that refutes the current BDNF hypothesis [50] that stress reduces the expression of BDNF and that antidepressants can reverse neuronal atrophy and this altered BDNF expression [51]. For example, levels of BDNF in the nucleus accumbens (NAc) and depressive-like behavior are increased in the stressed mouse [52]. The direct infusion of BDNF into the ventral tegmental area–NAc also increases depressive-related behaviors in the rat forced-swim test [53]. In addition, fluoxetine has either no effect [54], or even causes a decrease in BDNF mRNA in the rat hippocampus [55]. Recently, it was reported that BDNF gene polymorphisms are correlated with LDAEP [56], [57]. In particular, subjects with the Val/Met (A/G) genotype for rs6265, the T/T genotype for rs2030324, or the C/C genotype for rs1491850 had a higher LDAEP, indicating lower central serotonergic activity. A low LDAEP was more prevalent than a high LDAEP among those with the C-T haplotype [57]. Together these results suggest that the current theoretical formulation of the BDNF hypothesis is too simplistic, and hence BDNF-mediated signaling should be considered within a region-specific, antidepressant-specific, and genetic perspective [58]. Recently, the NMDA receptor antagonist ketamine exerts an antidepressant effect in patients with treatment-resistant MDD [59], [60]. Furthermore, ketamine rapidly reverses depressive behaviors and loss of neuronal connection [59]. However, these antidepressant effects of ketamine are blocked in BDNF-knockout mice [59]. Thus, BDNF also regulates NMDA receptor function and synaptic maturity [60].

It is intriguing that in the present study the high-BDNF subgroup had a more severe psychopathology, such as low central serotonergic activity, hopelessness, and impulsivity. The serotonin level decreases in individuals subject to acute stress [49], and it can be assumed that BDNF increases in an attempt to normalize this decreased serotonin level. Some investigators found that acute stress was associated with a decrease in the levels of serotonin mRNA in the dorsal raphe nucleus [49], [61]. In addition, rats subject to acute stress exhibit rapid increases in BDNF mRNA in the hippocampus, whereas the chronic stress leads to BDNF mRNA decreasing rapidly to levels significantly below that of normal controls [62]. Some investigators have recently reported the serum BDNF to be higher in patients with MDD than in healthy controls, although there was no significant difference [63]. Thus, BDNF levels in MDD remain controversial; more studies with larger samples are needed.

The small sample in this study may limit the generalizability of its findings. In addition, the sample did not include normal controls. Moreover, an evidence-based consensus was not used when stratifying the cohort into two subgroups according to depression severity; however, some clinicians consider that scores between 7 and 17 indicate mild depression [64]. Despite these limitations, this is the first study to demonstrate a relationship between BDNF levels and LDAEP in Asian depressive patients. In addition, an intriguing result was that the high-BDNF subgroup exhibited a more severe psychopathology in some psychometric rating scales or low central serotonergic activity, findings that conflict with previous results. More studies with larger samples should be performed to examine further the relationship between BDNF and central serotonergic activity in MDD.

## Supporting Information

Figure S1 Correlation between brain-derived neurotrophic factor (BDNF) levels and loudness dependence of auditory evoked potentials (LDAEP) (*p* = 0.025).
(PDF)

Figure S2 Correlation between BDNF levels and total Beck Hopelessness Scale (BHS) score (*p* = 0.016).
(PDF)

Figure S3 Correlation between BDNF levels and total Barratt Impulsiveness Scale (BIS) score (*p* = 0.067).
(PDF)
