# Supplementary figures and images for: Serum BDNF Levels in Relation to Illness Severity, Suicide Attempts, and Central Serotonin Activity in Patients with Major Depressive Disorder: A Pilot Study
Source: PLoS One. 2014 Mar 24;9(3):e91061. doi: 10.1371/journal.pone.0091061 (PMC3963843; doi:10.1371/journal.pone.0091061)

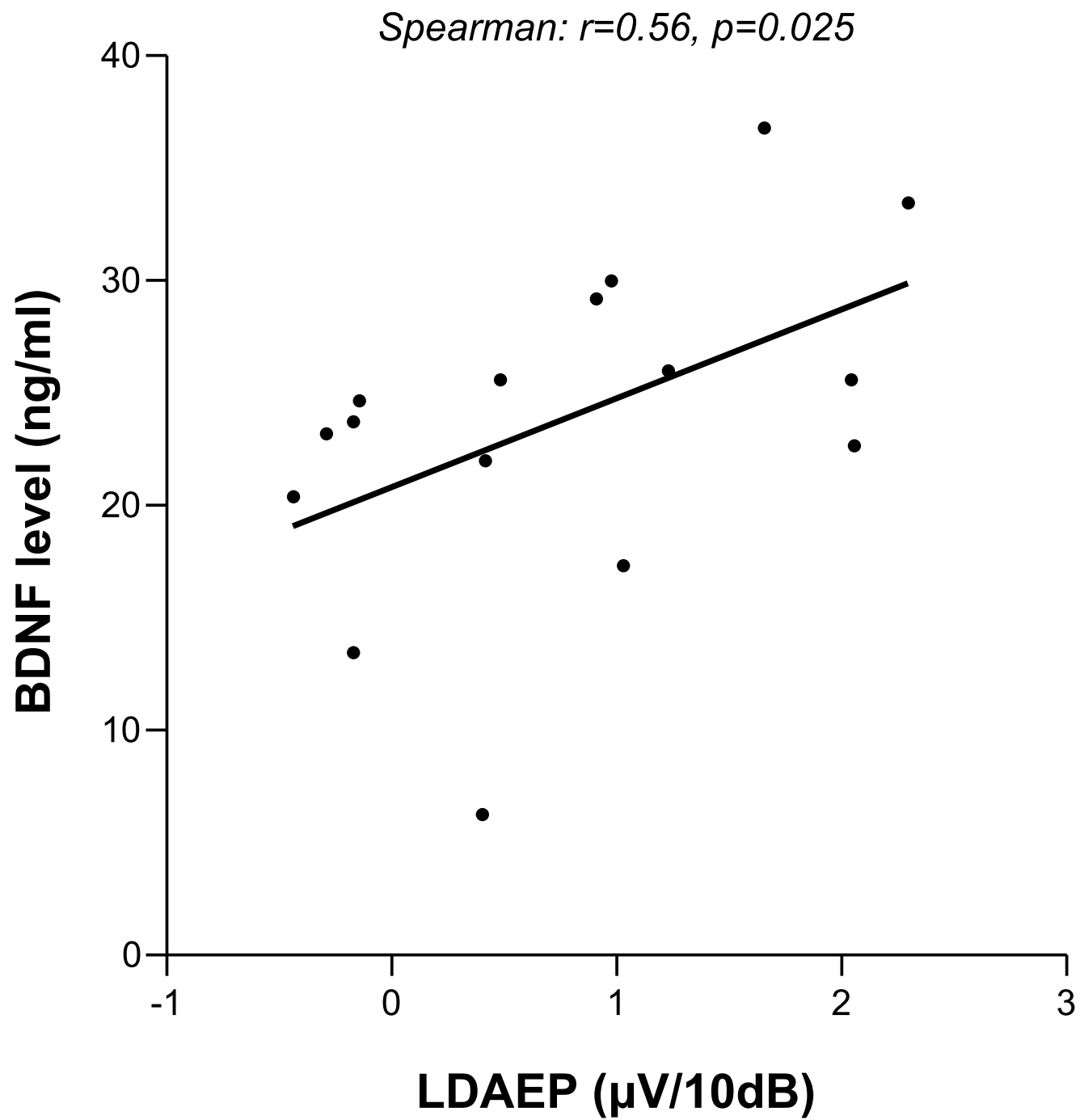

Supplement: Figure S1 — Correlation between brain-derived neurotrophic factor (BDNF) levels and loudness dependence of auditory evoked potentials (LDAEP) (p = 0.025). (PDF) [file pone.0091061.s001.pdf]

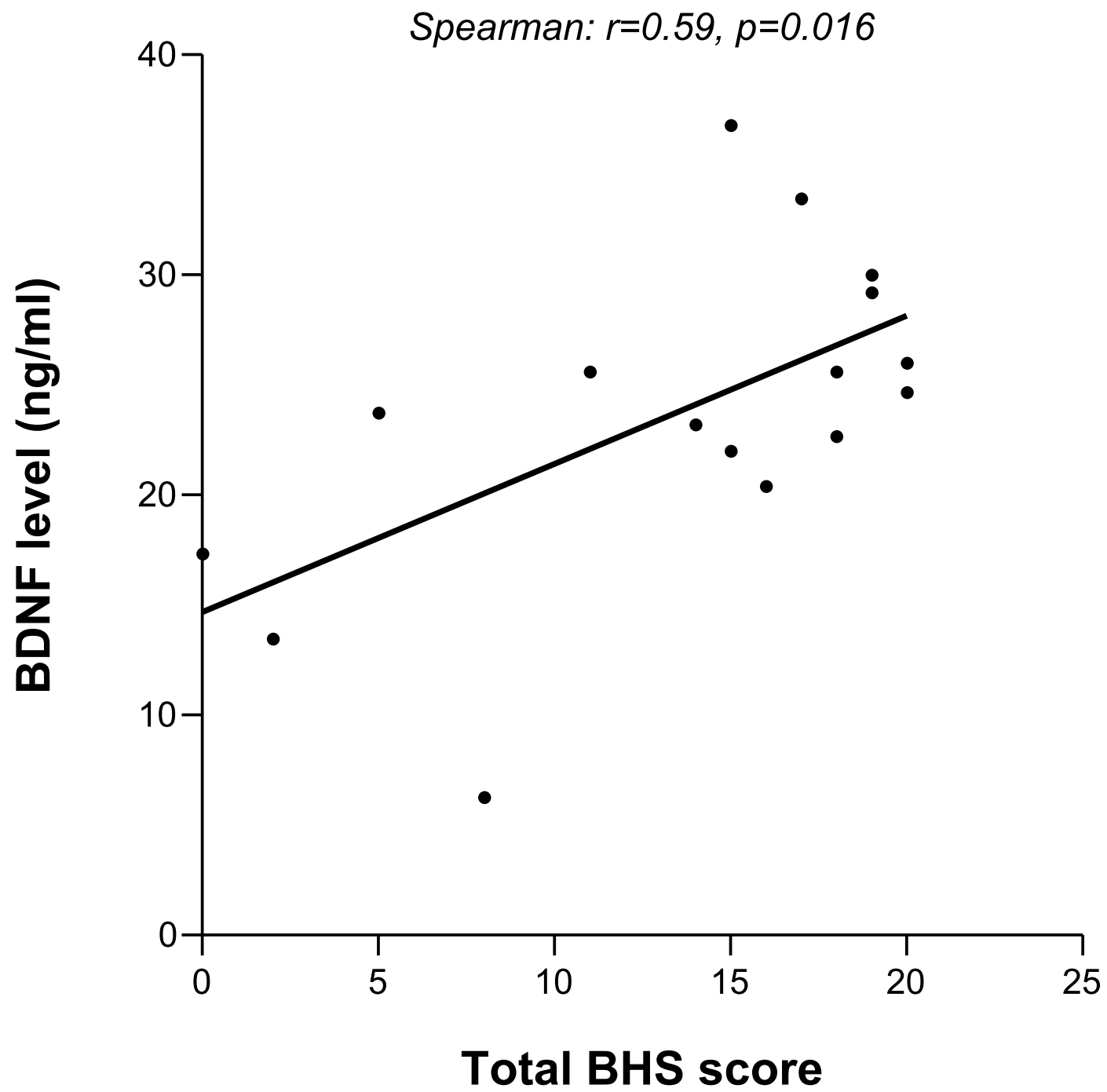

Supplement: Figure S2 — Correlation between BDNF levels and total Beck Hopelessness Scale (BHS) score (p = 0.016). (PDF) [file pone.0091061.s002.pdf]

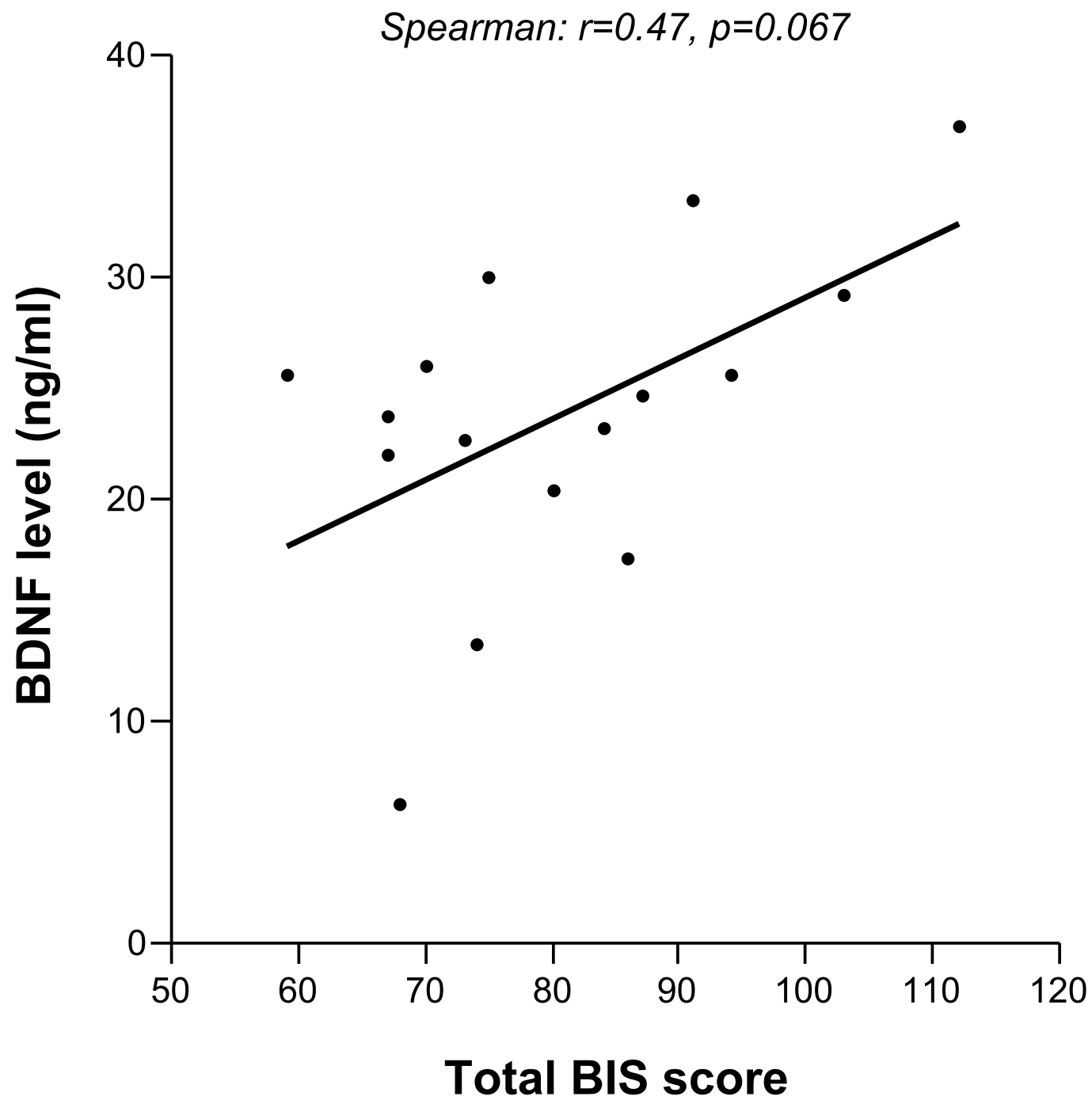

Supplement: Figure S3 — Correlation between BDNF levels and total Barratt Impulsiveness Scale (BIS) score (p = 0.067). (PDF) [file pone.0091061.s003.pdf]
